# Patterns of HIV testing practices among young gay and bisexual men living in Scotland: a qualitative study

**DOI:** 10.1186/s12889-017-4653-5

**Published:** 2017-08-17

**Authors:** Nicola Boydell, Katie Buston, Lisa Margaret McDaid

**Affiliations:** 1 Usher Institute of Population Health Sciences and Informatics, University of Edinburgh, Edinburgh, UK; 2 MRC/CSO Social and Public Health Sciences Unit, University of Glasgow, Glasgow, UK

**Keywords:** HIV testing, HIV prevention, Sexual behaviour, Qualitative, Young gay and bisexual men, MSM

## Abstract

**Background:**

Increasing overall rates, and frequency, of HIV testing in populations at risk is a key public health objective and a critical dimension of HIV prevention efforts. In the UK, men who have sex with men (MSM) remain one of the communities most at risk of HIV and, within this, young gay men are a key risk group. Understanding HIV testing practices is important in the development of interventions to promote testing among young gay and bisexual men.

**Methods:**

Qualitative interviews were conducted with thirty young gay and bisexual men (aged 18–29) in Scotland. Thematic analysis of men’s accounts of their approach to HIV testing identified three overarching patterns of testing: *‘habitual’, ‘reactive’ and ‘* *ad hoc’*.

**Results:**

This qualitative study, the first to explore patterns of HIV testing practices among young gay and bisexual men in the UK, contributes novel findings around the role of social support and ‘community’ in shaping young men’s approaches to HIV testing. The findings suggest that social support can play an important role in encouraging and facilitating HIV testing among young gay men, however, social norms of non-testing also have the potential to act as a barrier to development of a regular routine. Men with *habitual* testing practices framed HIV testing as both a personal and ‘community’ responsibility, and more effective than testing in response to risk events or emergent symptoms. Men who reported *reactive* testing practices described testing for HIV primarily in response to perceived exposure to sexual risk, along with ‘transitional moments’ such as starting, ending or changes to a relationship. Among young men who reported testing on an *ad hoc* basis, inconvenience and disruptions to HIV testing practices, particularly where men lacked social support, acted as a barrier to developing a routine of regular testing.

**Conclusions:**

Our findings suggest that interventions which seek to increase rates of HIV testing and testing frequency among young gay and bisexual men should include a specific focus on promoting and supporting positive testing practices within young men’s friendship groups and wider gay communities.

## Background

Increasing rates of HIV testing in populations most at risk of HIV is a key public health objective. Regular testing is an important part of HIV prevention efforts, and emphasis has been placed not only on increasing overall rates of HIV testing, but frequency of testing [1, 2]. Early diagnosis and subsequent treatment reduce an individual’s infectiousness and thus can have both individual and population level benefits [3]. Late diagnosis is a key factor in HIV mortality [4], and regular testing has a key role to play in both enabling access to early treatment and reducing onward transmission of HIV. The increasing focus on biobehavioural approaches to HIV prevention such as Treatment as Prevention (TasP) and Pre-Exposure Prophylaxis (PrEP) foreground the importance of regular testing within the ever changing HIV prevention landscape. Indeed, if PrEP is to be made routinely available it is likely that frequent testing, including self-testing, will play a critical role in ensuring success [3, 5–9].

In Scotland, and the rest of the UK, men who have sex with men^1^ (MSM) remain one of the groups most vulnerable to HIV [10]. Current UK policy on HIV testing recommends that MSM attend for testing at a minimum annually, but more often if engaging in high risk sex, specifically, unprotected anal intercourse^2^ (UAI) with multiple partners [10]. For those at highest risk of infection, UK national guidelines on safer sex suggest that it may be appropriate to (re)test as frequently as every 3 months [10, 11], however, there is little consensus on what is defined as ‘high(est) risk’ [12].

Increases in HIV testing have been widely reported in high income countries [13–19]. However, recent research in Scotland and the rest of the UK has shown that although HIV testing rates have been increasing among gay, bisexual and other MSM, increases in testing levelled off in the years 2008–2011 [20], and that testing frequency does not meet levels recommended in current guidelines [21, 22].

A recent study exploring HIV testing frequency among MSM in the UK identified variation in testing practices among young men (aged ≤25 years) with 38.6% reporting testing once or less in the past 2 years, and 27.5% reporting at least four HIV tests in the same period [21]. Furthermore, research exploring HIV testing histories among MSM in England found that when compared with those in their 20s, as a group, men under the age of 20 were more likely to have never tested for HIV [22]. This supports research from the Australian context which provides evidence that the proportion of those who report never testing is highest among young gay men, something the authors suggest is indicative of a lack of engagement with sexual health services among this group [23]. A recent sexual health needs assessment of MSM in central Scotland [24], concluded that younger men are highly sexually active, often have a high number of partners, and engage in high-risk sexual behaviour; factors which place them at greater risk of HIV. Many young men were less aware of HIV risk, and thus less equipped to employ strategies to reduce risk, including HIV testing [24].

Although a large body of research, both quantitative and qualitative, examining factors associated with HIV testing [25–27] and frequency and regularity of HIV testing among MSM [1, 16, 19, 21, 28, 29] exists, little work has explored young men’s testing practices. A more in-depth understanding of HIV testing practices among young MSM is crucial in developing strategies to increase uptake and frequency of testing, and to inform the development of interventions which seek to change testing practices among men in communities most at risk of HIV.

This qualitative study explored young gay and bisexual men’s understandings of HIV risk in the context of their sexual practice and community norms of safer sex. It sought to examine the role of people within men’s social and sexual networks in shaping and informing men’s approach to safer sex broadly, and HIV testing specifically. Here, we report on patterns of HIV testing among young gay and bisexual men living in Scotland, with a specific focus on the role of social support and ‘community’, and explore the implications for interventions to encourage uptake, and increase frequency, of HIV testing.

## Methods

Semi-structured interviews were conducted between October 2012 and July 2013 with 30 young gay and bisexual men living in Scotland. Ethical approval was obtained from the University of Glasgow College of Social Sciences Ethics Committee [Application number: CSS20120206].

Key inclusion criteria were that the men were aged between the ages of 18 and 29 years and identified as gay, bisexual, or as a man who has sex with other men. We sought to recruit men from urban, semi-urban, and more rural areas of Scotland. Varied recruitment strategies were used: flyers, posters and digital advertisements distributed through sexual health organisations and support groups working with gay and bisexual men; professional networks; snowballing; and via social media such as Twitter and Facebook.

Participants were predominantly White Scottish. Two men were from Ireland, and two were from outside the UK, however, all had been resident in Scotland for more than 4 years at the time of interview. No men from Black, Asian, and minority ethnic communities participated in the research. All but one of the men who participated in the study identified as gay, with one describing himself as queer, and noting that in the past he would have identified variously as bisexual or gay. Another participant described himself as gay during the interview, but noted that in other contexts he might describe himself as bisexual or ‘questioning’.

The majority of men interviewed lived in, or close to, three main urban centres, with the remainder from four more rural, regions of Scotland. Nine were in coupled relationships with other men, and reported relationship lengths between 3 months and just over 5 years. All but one of the men were HIV negative or undisclosed. Given the focus on *current* HIV testing practices, the account of the HIV positive participant was excluded from this analysis. Table 1 outlines key demographic characteristics of the sample.

**Table 1:** Demographic characteristics of study participants

Participant characteristics	
	Number of participants
Age range (Mean Age = 23)	
18–24	18
25–29	11
Education (highest level completed)	
School leaving qual. (e.g. ‘O’ levels or CSEs and A-level or Higher qualifications)	4
Further education qualification (e.g. NVQ, SVQ, HNC),	11
University degree	11
Declined to provide educational qualifications	3
Employment status	
Employed (full or part time)	10
Unemployed	8
In education (full or part time)	11

Interviews ranged in length from 40 to 140 min, and typically lasted 1.5 h. Participants’ provided written informed consent. Topics covered included: men’s social networks and communities; understandings of safer sex; safer sex in the context of sexual practice; and HIV risk, and testing practices.

Interviews were digitally recorded, transcribed verbatim, anonymised, assigned pseudonyms, and entered into NVivo 9 for data management and coding. A thematic approach to analysis was used; interview data were coded and charted using principles of the Framework approach [30, 31]. The first author developed a coding framework based on both predetermined research questions and themes identified through close reading of interview transcripts. The second and third authors read a selection of transcripts, and reviewed the coding framework. The coding framework was applied to all transcripts; this enabled systematic comparisons to be made across the data and facilitated the identification of recurrent themes [32]. Coding was reviewed regularly by all three authors to ensure emergent issues were incorporated into analysis. In line with the Framework approach, and qualitative approaches more broadly, specific attention was paid to contradictory or divergent cases [32, 33].

## Results

HIV testing was discussed by all participants, and all reported testing for HIV at least once. A key theme across the men’s accounts was the important role of sexual health screening broadly, and HIV testing specifically, as part of the range of safer sex and risk management strategies employed in their sexual lives. Regardless of their own approach to HIV testing, virtually all men described being aware of the need to test and many emphasised the benefits of testing, both individually and for wider gay communities. Nevertheless, analysis of participant’s accounts emphasised the complexity of patterns of HIV testing. We identified three overarching patterns of HIV testing practices across the men’s accounts; *habitual, reactive* and *ad hoc*. Although the men did not define approaches to testing using this terminology, these categories are grounded in the men’s accounts and are intended to illustrate the patterns observed and developed through the analysis.

### Habitual testing practices

Just over a third (*n* = 12) of the sample described their testing practices in a way which could be conceptualised as habitual. They emphasised the importance of regularity of testing, with all reporting attending for HIV testing at least every 6 months, although some reported testing more frequently. For the majority of this group, a pattern of regular testing had started early in their sexual lives and was well integrated into their approach to HIV risk management and safer sex practice. Most acknowledged that there was a heightened sensitivity to, and awareness of, HIV among gay communities. As Theo observed:

With gay men anyway, I don't think there's any kind of group within that that can say, apart from people that are abstinent, to say, you know, “we're not at risk [of HIV], it's not an issue for us”. It's definitely a community thing. (Theo, 23)

Regular screening for HIV and other sexual transmitted infections (STIs) was framed as a more successful approach to maintaining good sexual health than testing in response to risk events (e.g., condomless anal intercourse (CAI) with a casual partner or partner of unknown status) or emergent symptoms. Indeed, accounts typically focused on regular HIV testing as an integral part of leading a responsible gay sexual life and the men framed their testing practices within an ethic of care; a responsibility for self-care, care of potential sexual partners, and towards men in their wider communities in encouraging them to test as part of a regular routine. As Tom explained:

It’s all precautionary and being responsible [...] And I tell my friends about it, like I, you know, if I go and test I like Tweet about it and I get, we call it our MOT and, a lot of people do. (Tom, 26)

Similarly, Tiernan, talking about his work on reception at a sauna on the commercial gay scene, described not only his own commitment to regular testing, but what he described as his sense of responsibility to the ‘gay community’ to encourage men he met in the course of his work to test. As he explained:

I know a lot of people in the sauna, and if I get to know people well enough that I can have a laugh and joke with them and stuff, I'll ask them. I'll go, “did you go for a sexual health check recently?” [...] kind of, in a joking fashion, but you're kind of getting them to think**,** and they do think about it. I have got people who were like, “oh, I went for my sexual health test and stuff and was clean”. And I was like, “that's good, wasn't difficult, was it?”, and kind of... you're kind of reaffirming it yourself, and as I say, I consider myself a part of the gay community, so even by me just issuing or kind of saying these things by reaffirming stuff I am spreading the message. (Tiernan, 25)

Open communication about sexual health, specifically the topic of HIV, was perceived as playing an important role in encouraging regular testing among the men and their friends. Among this group, high proximity to HIV was reported. For example, knowing people living with HIV or being in (or having considered starting) a relationship with an HIV positive partner. Although men acknowledged that stigma made discussion of HIV problematic in some circumstances, there was minimal anxiety or stigma around discussing the topic, at least among their close friends.

The importance of support and encouragement from friends to access testing or actually attending with friends (both male and female) for testing was emphasised. Indeed, open discussion around HIV (and other STIs) with close friends appeared to enable men to make HIV testing a more ‘social’ event. This social dimension of testing was highlighted in a number of accounts. As David explained:

When we [group of close friends] ever had our 6 month or 3 month check-up, we made a day of it, so we’d go to the GUM clinic in the morning and then we’d go for lunch afterwards and we’d be like, “oh, yay, we’re all clear, everything’s fine!” that kind of thing, so...Make it into an event rather than a ‘oh my gosh, I’m so worried,’ so we’d actually... And from that, we think that we’ve never really had an issue with getting ourselves tested, because I think as gay men as well, we are always told, “get yourself tested, get yourself tested”. (David, 27)

The social support David and his friends received from each other around testing appeared to allay some of their anxieties about testing individually, and reinforced their commitment to caring for themselves, their close friends, and wider communities of gay men.

It is important to note that men who described having habitual testing practices did report instances of testing in response to perceived risk events; for example CAI with a casual partner of unknown status, or after partner notification of an STI. Nevertheless, these testing events were framed as being *in addition to* their regular screening. Indeed, where potential sexual risks events were identified, some suggested that the routine nature of their HIV testing practices meant that they would have an early diagnosis, thus gaining access to treatment and support, both from clinicians and friends and family. As Noel explained:

...if I did ever get it [HIV], I know I because I get tested for it, I know I’m in the right hands and I know I’m going to get the right support for it. [...] As long as I got myself seen to, made sure I got myself on the proper medication, got the proper support about it, got myself a proper diet, made sure I’m doing everything the right way and I’d be ok. (Noel, 23)

### Reactive testing practices

Another third of the men (*n* = 9) in the sample described their testing practices as reactive, being primarily motivated by a perceived exposure to HIV risk. They cited a range of factors that motivated them to access HIV testing: episode(s) of condomless or ‘unprotected’ sex; symptoms of infection; higher numbers of sexual partners; and what could be described as ‘transitional moments’ such as starting, ending or changes to a relationship.

Although this group recognised that HIV screening was recommended and an important aspect of risk management and safer sex practice, they did not emphasise regularity of testing within their accounts nor describe maintaining a regular routine of self-care. Men talked about how changes to the ‘status’ of an intimate relationship acted as a motivation for testing. Similar to men with habitual testing practices, men often articulated a sense of responsibility to themselves and their partner to test for HIV:

After me and my partner broke up, we both just said, I was like that, “look – I believe that you haven’t, you know, done anything with anyone else. I know that I haven’t. But I would much rather that we got [tested]” – we both just agreed that we’d go and get checked. I said “look, we’ll need to do it anyway”, I says, “so let’s just go and do it together”. And we did. (Colin, 24)

Another motivation for accessing testing described by participants were episodes of condomless sex. For example, Tony talked about his early experiences of sex, which did not involve use of condoms, and his realisation that this may have exposed him to risk of HIV and other STIs. Tony responded to this perceived risk exposure by attending for testing at his local genitourinary medicine clinic:

The first time I went to the GUM clinic I was 15, 16 […] I didn’t use a condom the first time. Or a few times after that, either. I kind of put that two and two together through [watching] TV. Basically. So it made realise I needed to go and get checked. (Tony, 21)

Although at an individual level men in this group understood HIV as a potential risk associated with sexual behaviours such as CAI, in contrast to men with habitual testing practices, the younger men in this group (*n* = 6), those aged 24 or under, expressed the view that less emphasis should be placed on HIV transmission among communities of gay men:

HIV is a risk for everyone... I don’t think it should just be stigma’d to gay people because it was, I’m sure it was like… the statistics were like women had it more than gay men just a few months back, a few years back maybe. It’s not a specific issue for the gay community. I think it’s a specific issue for everyone, everyone, because anyone could have it. It’s not just a certain category. It’s anyone can have it. (Ed, 20)

This particular group of younger men appeared to view HIV risk, and responses to it, as more individualised, resisting the notion of HIV as a ‘community’ or collective issue.

Men who described reactive approaches to HIV testing rarely described knowing people living with HIV. Their accounts suggested that HIV was relatively removed, or distant, from their everyday experience, and something which they felt they did not have to respond to regularly in social, or indeed sexual, contexts. This is illustrated by an extract from Kalen’s account:

I have thought before about what I would do if I was on a date with someone and they, they said “I’m HIV positive”, and I have no idea […] I don’t know how I would react in that situation. It’s not something, I don’t know anyone with HIV, I don’t, I don’t know of anyone who has HIV, in, in my social life or, or that I’ve met personally, so I don’t think of it as *something.* (Kalen, 29)

Kalen’s reflections around the possibility of a ‘date’ disclosing that they were living with HIV emphasises how his sense of distance from HIV, socially and sexually, made it more difficult for him to imagine how he would respond. Moreover, HIV was not widely framed as a topic of discussion with potential sexual partners, nor people within their close friendship groups and wider communities. Discussing HIV was framed as problematic because of the stigma associated with the condition and as Kalen (29) articulated, as running counter to the norms of “gay society”. A number of men emphasised their anxiety that raising the topic of HIV could be interpreted by close friends, sexual partners, and wider gay communities, as an indication that they were at risk of HIV.

Lack of open and easy communication around HIV also appeared to translate into fewer opportunities for being supported, or encouraging others, to access HIV testing. Among men who described reactive testing practices, only one reported being supported to go for testing by a friend, and then only after an episode of condomless sex with a casual partner:

One night, me and this guy had just got drunk and slept together, and I was just kind of like, ‘oh no’. And I needed to go and get checked – and like, I was just, like, ‘oh my God’. I talked to my flat-mate, about it because they’d had experience of STIs. And I was, I was kind of shitting it, and they were just like, “no, you’ll be fine”. They came up with me and took me to get tested and stuff. (Colin, 24)

None of the other men described testing together with friends, or indeed being encouraged to do so by friends or those within their social networks. Thus, for the majority of these men, testing appeared to be highly individualised, not embedded as a more social practice within their friendship groups or the communities in which they were situated.

### *Ad Hoc* testing practices

The remainder of the sample (*n* = 8) described HIV testing practices in ways which did not follow the patterns described thus far. This group of men described *ad hoc* approaches to testing. Men with *ad hoc* approaches to testing described proactively seeking opportunities to test outside the context of a risk event, and they differed from those who described a reactive approach to testing in that they accessed testing because they believed they *should*, not primarily in response to exposure to HIV risk. Their HIV testing practices also differed from men with a habitual approach in that, despite being motivated to test, they had not established a regular routine, or this had been disrupted in some way.

Some of the group of men with *ad hoc* testing practices described how their approach to testing had changed over time, and their accounts provide insights into factors that may act to ‘push’ and ‘pull’ men between different practices. For example, Quentin described previously having a habit of regular testing which had been disrupted. When he was younger his close friendship with an HIV positive friend and other peers, combined with encouragement from an influential teacher, had acted as a catalyst, or push, to develop a regular routine of testing at his local medical practice. In contrast, he currently tested infrequently and attributed this change to difficulties in accessing sexual health services and social support for testing in the rural area he had moved to. Although he noted that there were a variety options for accessing testing, such as at a local sexual health service, in his experience the staff there made this difficult:

...they’ve got the [name of service] but the dragons who work in there, they just make you feel guilty for going in. They make you feel guilty for going in [...] I went in and they just made me feel uncomfortable just sitting there waiting to see someone, do you know what I mean? So if I’m feeling uncomfortable how is someone else gonna feel? They don’t seem to realise that. (Quentin, 29)

This appeared to be a pull factor, and barrier, to accessing testing for him and he recognised that this could also represent a barrier to testing for other men. Quentin had recently started volunteering for a gay men’s charity, and anticipated that this would open up more possibilities for supporting, and being supported, to attend for HIV testing in alternative community-based settings.

Men with *ad hoc* testing practices who grew up in rural areas some distance from the main urban commercial scenes in Scotland (which are primarily concentrated within the cities of Glasgow and Edinburgh) often reported gravitating to online communities and spaces when seeking social and sexual connections with other gay men. In the absence of easy access to a physical gay community (such as bars, clubs and saunas part on the commercial gay scene), sociosexual media and online networks (i.e., use of a variety of online LGBT forums, Gaydar, Grindr etc.) were important connections to gay communities. Nevertheless, men were clear that these online connections did not necessarily support them to develop regular HIV testing practices in that they did not present opportunities for *active* encouragement to attend testing, or indeed to test with friends or peers. This, combined with lack of easy access to sexual health services, in particular community-based clinics, was cited by these men as making it more difficult to attend for regular testing. As Terry explained:

I spoke to the people on the instant messenger thing [online outreach worker] and it was also about getting tested for sexually transmitted diseases, kind of had a bit of a sort of brainwave, l should go and get this tested. And I spoke to, and they kind of referred me to go to a place up in [name of city]. So yeah, that was, I think it was only like once it was that I was going to get done there. (Terry, 27)

The community-based clinic referred to by Terry was in a city some 25 miles away and, as he explained, this made it difficult for him to return for regular testing. Despite proactively seeking information about accessing testing in online settings, this group’s accounts suggest that access to sexual health services for gay men may be more problematic - or at least appear more problematic - in rural areas, and thus a contributory factor in not developing a pattern of regular testing.

In contrast to men with habitual testing practices who emphasised the positive role that support from friends played in encouraging them to access HIV testing, some men with *ad hoc* approaches noted that social norms could contribute to *not* developing a regular routine of testing. For example, despite acknowledging public health recommendations that gay men should test regularly, Nick suggested that his friendship group, comprised predominantly of heterosexual men, tended not to emphasise the value of HIV and STI testing. He explained:

...you know, the ‘football guys’ are my core group, if there was also a ‘gay group’ or, or an offshoot of one of those that was, that was composed of gay men, I think you would have different conversations and I, feel like I would benefit from their input on things [...]I don’t get regular health check-ups or STD testing from like a gay men’s health clinic or whatever, which is something I think I should do, but I don’t and again, it’s easy for me not to because I don’t really know anyone else that’s doing that. So I don’t have a routine of doing that or, or, whatever, and I know that I should but again, it’s something that never comes, it never comes up in conversation with my, almost exclusively straight, circle of friends. (Nick, 29)

Thus, while men in this group acknowledged their awareness of HIV testing recommendations for gay men this did not necessarily translate into the practice of habitual testing for them individually. Indeed, differential social norms of testing, access to services, and the availability of supportive resources affected, and at times disrupted, participants HIV testing practices.

## Discussion

This is the first qualitative study to explore patterns of HIV testing practices among young gay and bisexual men in the UK. Our research provides insight into factors that shaped and informed men’s approaches to HIV testing; those which served to facilitate the development of regular testing habits, and those which served to discourage or disrupt the development of such routines.

Contrasting explanations for the role of social norms in the development of testing practices were apparent. Men with habitual testing practices reported more instances of *active* support to attend for testing from people within their friendship groups, and the communities in which they were embedded, than those who described reactive or *ad hoc* practices. They emphasised that going for testing was the norm among their close friends and sexual partners. Moreover, some were encouraged to test, not only on the basis of open discussion of HIV, but by practical (or *active)* support including actually attending for testing together. This more social dimension of HIV testing differed from the accounts of reactive testers who reported little discussion of HIV testing, which appeared to translate into few opportunities for testing with friends and peers. Men who described *ad hoc* testing practices emphasised that lack of community and social support, and social norms around non-testing could serve as a disincentive in developing a regular routine of testing.

To a certain extent our findings echo those of Arnold and colleagues [29] who explored HIV stigma as a barrier to testing. Young men in our study who reported reactive testing practices voiced a concern about stigma in discussing HIV (and HIV testing). The way in which men under the age of 24 in this group sought to distance both themselves, and wider gay communities, from being linked to HIV risk suggests that HIV stigma, combined with lack of personal connection to people living with HIV, compounded their anxiety about open communication around HIV status and testing. Indeed, it was more common for men with habitual testing practices to report knowing someone living with HIV, and to describe having open discussions around HIV, than it was among those describing reactive approaches. Previous research suggests that, among men with high proximity to HIV, HIV had a social dimension and that discussion of HIV, including disclosure of HIV positive status, did not violate social norms [34].

Given the importance of social support discussed by men with habitual testing practices, it may be that strategies appealing to friends to support or advocate for testing together may be a fruitful avenue to consider in developing future interventions. Interventions to increase HIV testing among young men should include components designed to build on social support among men, and increase resilience [35]. It is important that men continue to be supported and encouraged, both individually and at community-level, to have open conversations about HIV testing and re-testing, and the value of knowing their own HIV status as a part of the process of sexual decision making [24]. Although many HIV organisations in the UK, and internationally, already promote the importance of open conversations with peers about HIV testing, the fact that some men described the particular value of *active* support from friends to attend for testing together, suggests that promotion of this approach should be incorporated into ongoing efforts to normalise testing. This may help to support, and increase, regularity of testing.

Although the small sample size makes it difficult to be definitive, men who lived in rural areas described support for, and access to, HIV testing services as more problematic than the majority of men living in urban areas. That one participant, Quentin, who described a previous habitual pattern of testing emphasised the role of negative staff attitudes and lack of social and community support in disrupting his routine of regular HIV testing speaks to the idea that service provision in some rural areas may not be perceived as fully meeting the needs of MSM. This highlights the importance of provision of ‘gay friendly’ community-based sexual health services that meet the needs of young men [23]. Further research could usefully explore the experiences of men in rural areas with a view to informing service provision and access to community-based testing services.

Another issue to consider, particularly in relation to men who described reactive testing practices, is the period after potential exposure to HIV infection and before markers of infection are detectable; the ‘window period’ [34, 36, 37]. Current UK guidelines recommend HIV testing using fourth generation laboratory HIV test (which test for HIV-specific antigen and antibodies simultaneously) [34, 37] as these will detect the majority of infections at 4 weeks after exposure. Newer ‘rapid’ tests have a longer window period [36]. Although some participants did make reference to their awareness of HIV testing window period, this was not discussed in depth. This issue may be particularly problematic for men who described reactive testing practices. Where men described testing in relation to a particular risk event, it is possible that test results may not necessarily reflect the particular event they were testing for. This highlights the need for health promoters and practitioners to use attendance for HIV testing as an opportunity for discussion of ongoing risks for HIV infection and the need to re-test regularly.

In contrast to Hussen and colleagues [27], none of the participants in this study could be categorised as ‘test avoiders’ for whom fear about the implications of receiving an HIV positive diagnosis - including stigma, community rejection, and illness/death - were key barriers to testing. Although some men who described reactive and *ad hoc* testing practices may at times have been *reluctant* testers, they had all previously attended for testing and planned to do so again in the future. It is possible that men in our study considered themselves at low risk of HIV and were overall less anxious about HIV testing, and indeed the possibility of receiving an HIV positive diagnosis, in contrast to the study above, which recruited men reporting at least one instance of unprotected anal intercourse in the previous 6 months [27].

Our findings do suggest that testing practices are not fixed, echoing Hussen and colleagues [27] who suggest that people move between testing behaviours. Although the majority of men with habitual testing practices reported having a consistent approach to STI and HIV screening, a number of men with reactive and *ad hoc* patterns of HIV testing described changes in their approach over time. Others described aspirations to change their testing practices in future, with some intending to test more frequently or test with future sexual partners. This is important in terms of developing interventions which seek to increase frequency of testing, as changes in social and sexual contexts may facilitate a shift in men’s approaches to HIV testing over time. Indeed, strategies designed to facilitate regular testing must take into account individual perceptions of HIV risk, the social and sexual contexts in which such responses to risk are developed, particularly within gay communities, as well as with structural issues such as stigma and marginalisation.

It is important to consider how discourses of responsibilisation around HIV testing permeate the findings. Given that the sampling criteria were not based on assessment of sexual risk behaviours, it is worth noting that participants were not necessarily ‘high risk’ young men. Many participants did not perceive themselves as personally being at high risk of HIV infection, and as such tested for reasons other than those advocated in testing guidelines for those at greatest risk of HIV [10, 11]. Indeed, participants’ accounts suggest that men were aware of expectations for self-monitoring and embodied risk management practices, which related to being at risk of HIV in public health and epidemiological terms [38, 39–41]. Regardless of their own approach to testing, all participants articulated awareness of HIV testing as a social norm, if not a moral imperative, for gay men and can be interpreted as recognition of the requirement to be a responsible sexual citizen [38, 40, 41]. The findings suggest that social norms around HIV prevention shaped men’s understandings of testing as an obligation, particularly among those with habitual testing practices, and acted as a strong motivation to test regularly. Participants described their understandings of the need to test as a way of protecting both their own health and that of their sexual partners and wider community. This speaks to their understandings of social norms of HIV risk management in terms of their relationship to an “imagined biosocial community” of men vulnerable to, and affected by, HIV [36]. Moreover, while HIV testing was recognised as an individual bodily practice some participants described the importance of testing together, thereby transforming the practice from an individualised, or medicalised event, into a more ‘social’ event.

It is critical that researchers continue to be attuned to the way in which changes in the social and sexual world that gay men inhabit, including the increasing focus on pharmaceutical HIV prevention technologies, shift understandings of risk and responsibility, and imbue HIV testing with new meanings [7]. As Keogh and Dodds’ [7] note, as individuals and communities engage with ‘new’ HIV prevention technologies, those involved in health promotion and intervention design must continue to engage with earlier research that focused on issues around morality, responsibility and rights, with a view to ensuring that ongoing prevention efforts are equitable and do not increase health inequalities among gay, bisexual and other men who have sex with men.

Particular strengths of this study include: the generation of rich qualitative data which provides insight into the diversity of ways in which young men positioned themselves and their testing practices within ‘community narratives’ around HIV testing [42]; and the focus on young gay and bisexual men living in Scotland beyond areas of high HIV prevalence, which are less well researched. However, the study has some limitations. This was an exploratory qualitative research study with a relatively small sample of young men, therefore generalising the findings from the study to other groups may not be possible, particularly those with different cultural expectations of social and community support, and health services. Another limitation relates to the rapidly changing HIV prevention landscape. Participants’ accounts of their HIV testing practices and risk management strategies necessarily reflect their experiences in the specific temporal period in which data were collected. Indeed, understandings of the role of HIV testing in prevention are open to rapid change, especially given the increasing focus on biobehavioural prevention strategies. Furthermore, half of the young men who participated in the research were recruited (directly or indirectly) through community-based organisations, organisations whose remit includes the promotion of HIV testing, and this is likely to have shaped participants understandings of social expectations and community norms relating to HIV testing. Since gay communities are changing [43] in response to a variety of factors, not least social and sexual media and development of online communities, community narratives around HIV testing could usefully be explored in further research.

In general, the young men who participated in the study reported high levels of sexual health knowledge, specifically around the transmission of HIV and other STIs. This group of men also described having high levels of social and familial support. Young gay and bisexual men are a group who may lack social support and have low levels of sexual health literacy [24, 44], so it is possible that this particular sample differs from the wider MSM population, both in the UK and internationally. Furthermore, all participants reported attending for HIV testing (and STI screening more broadly) at some point in their lives. Although this is similar to findings around STI screening from a bar-based survey of gay and bisexual men in Scotland [12], it may be that this sample of young gay and bisexual men is not representative of the wider population of gay men living in Scotland.

## Conclusions

This study suggests that young men’s HIV testing practices are shaped and informed by individual, interpersonal, community, and wider social contexts. Social support (or lack of) among friends, peers and wider communities of gay men has an important role in shaping men’s testing practices. Interventions which seek to increase rates of HIV testing, and testing frequency, could include a specific focus on promoting and supporting positive testing practices within young men’s friendship groups and wider gay communities. Such work would benefit from further understanding the role of social networks and community norms in promoting HIV testing and in re-examining peer-led approaches to HIV prevention, while continuing to pay attention to shifting understandings of risk and responsibility that continue to imbue HIV testing with new meanings.

## Abbreviations

CAI: Condomless anal intercourse
LGBT: Lesbian, gay, bisexual and transgender
MSM: Men who have sex with men
PrEP: Pre-exposure prophylaxis
STI: Sexually transmitted infection
TasP: Treatment as prevention
UAI: Unprotected anal intercourse

## Footnotes

1 The term MSM is used widely within public health (policy and wider academic literature) as a way of referring to all men who have sex with other men, regardless of whether or not they identify as gay or bisexual. As Mustanski and colleagues note [41] one reason for using the term MSM is that it refers to behaviour, not identity, however, this has been criticised for ignoring social aspects of sexuality. In the introduction and discussion sections, we use the terms gay, bisexual, and MSM where these terms are used in the literature cited. In the methods, findings and discussion sections we use the term gay, bisexual and, to a lesser extent, queer, to reflect the language participants used when talking about themselves and others.

2 In this instance we have used the term unprotected anal intercourse (UAI) as this is indicative of the language used in the guidelines cited, however, throughout the rest of the article we use the term condomless anal intercourse (CAI) as this better reflects current usage and sexual practices in the context of biomedical options for HIV prevention.
